# Design and SAR assessment of three compact 5G antenna arrays

**DOI:** 10.1038/s41598-021-00679-8

**Published:** 2021-10-28

**Authors:** A. Lak, Z. Adelpour, H. Oraizi, N. Parhizgar

**Affiliations:** 1 Department of Electrical Engineering, Shiraz Branch, Islamic Azad University, Shiraz, Iran; 2 School of Electrical Engineering, Iran University of Science and Technology, Tehran, Iran

**Keywords:** Health care, Engineering

## Abstract

In this paper three different multi stub antenna arrays at 27–29.5 GHz are designed. The proposed antenna arrays consist of eight single elements. The structure of feeding parts is the same but the radiation elements are different. The feeding network for array is an eight way Wilkinson power divider (WPD). To guarantee the simulation results, one of the proposed structures is fabricated and measured (namely the characteristics of S_11_, E-, and H-plane patterns) which shows acceptable consistency with measurement results. The simulation results by CST and HFSS show reasonable agreement for reflection coefficient and radiation patterns in the E- and H- planes. The overall size of the proposed antenna in maximum case is 29.5 mm × 52 mm ×  0.38 mm  (2.8 ${{\varvec{\lambda}}}_{0}$× 4.86${{\varvec{\lambda}}}_{0}$ × 0.036${{\varvec{\lambda}}}_{0}$). Moreover, for Specific Absorption Rate (SAR) estimation, a three-layer spherical human head model (skin, skull, and the brain) is placed next to the arrays as the exposure source. The simulation results show that the performance of proposed antennas as low-SAR sources makes them ideal candidates for the safe usage and lack of impact of millimeter waves (mmW) on the human health. In all three cases of SAR simulations the value of SAR_1g_ and SAR_10g_ are below the standard limitations.

## Introduction

Recently the 5G technology has become an attractive subject in the telecommunication industry. Upcoming 5G systems should satisfy several requirements such as: higher bandwidth, low latency, broad coverage of network, high reliability, high throughput, high connection density, low power consumption, high gain^1^. Some frequency bands have been proposed as candidates for millimeter wave (mmW) for example 27–29.5 GHz, 36–40 GHz, 47.2–50.2 GHz^2^. High path loss owing to reduced size of antenna dimenssions and increasing atmospheric absorption are two problems at high frequency. Although higher data rates can support by these frequency bands but the signal wavelength becomes shorter and according to the Friis equation, the free space path loss becomes higher^3,4^. Imployment of high gain directive antennas or antenna array is a solution to compensate such problems, which provides multipath supperssion and interference mitigation however low radiation toward human tissues is expected to achive low specific absorption rate^5^.

The 5G antennas usually use in handheld devices, such as tablets and mobile phone therefore they evidently should be small in size and light weight. It has been demonstrated when the radiation patterrn of antenna is directed to the top or bottom edges of the devices (that is endfire pattern) the influence of user’s hand on the antenna radiation is minimize^6^. Antenna arrays at 5G systems can be designed by some technologies such as microstrip and SIW^7,8^, and in many types like fermi, vivaldi, quasi yagi, and cavity backed^9–11^. However, the effects of electromagnetic field on human body tissue should evaluate by possible methods like numerical methods to ensure that these field sources do not threaten human health at 5G frequency bands. To appraise the exposure some parameters use by standard institutes such as Specific Absorption Rate (SAR), power density (PD), and the Skin Surface Temperature Elevation. There are some standards, such as Federal Communications Commission (FCC) and IEEE to determine the permissable values of SAR from exposure to electromagnetic fields for human safety. Their values are different for occupational and public environments. According to these standards the SAR_1g_ and SAR_10g_ limits are 1.6 W/kg and 2 W/kg repectively^12^.

The studies about SAR levels on human tissues have been done in many vaious conditions and methods such as in vivo–in vitro environment and also by numerical methods. Duo to the probable hazards on human health in actual conditions, many assesments about field exposure are conducted by software simulations and exprimental environments. In^13^, for the determination of SAR, the human body tissues are modeled in one (skin) and three layer (including skin, fat, and muscle) and a four-element array of rectangular patch antenna as an exposure source have been modeled by the CST softawre. The input powers were 20 dBm and 24 dBm and the frequencies were 28, 40 and 60 GHz. The results showed that at both power, SAR_1g_ and point SAR values at 28 GHz were lower than other frequencies^14^, the penertation of radiation at 30 GHz in human ear canal and tympanic membrane have been investigated and the results showed a very low penetration and not notable significant thermal effect on the tympanic membrane. In^15^ the absorption of RF field at 39 GHz both in invivo bovine the brain tissue and a brain simulating gel model have been investigated. The results represented the SAR and radiation penetration in the brain model, and therefor SAR, decreases with increasing depth and frequency. In^16^ the SAR values in head model of children and adults at 28 GHz (30 mW) and a microstrip antenna as a field source have been simulated. The results showed that absorption in tissues decreasing rapidly in depth. As well as duo to epidermis and dermis thickness (0.1 and 2 mm), the mmW values values are quickly absorbed in these layers and do not reach the deeper tissues.In this paper three compact, lightweight, high gain eight arrays antenna are simulated at 27–29.5 GHz. Design procedure, simulation, and measurement results are presented in the following sections. Also the SAR_1g_ and SAR_10g_ have been simulated and evaluated to determine the specific absorption rate.

## Antenna design

### Feeding part

**Figure 1:**
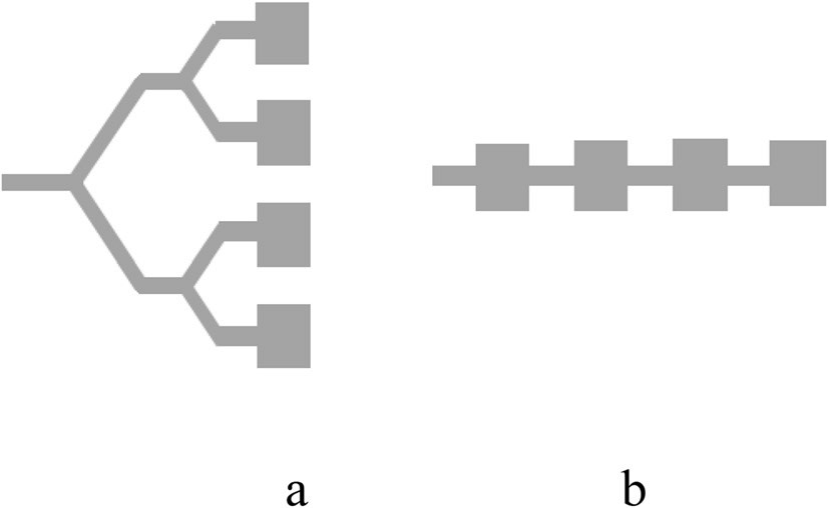
(**a**) Corporate and (**b**) series feed array structure^17^.

There are different types of feed network for feeding an array antenna. The formal array feeding networks are series or corporate feed network based on microstrip structures^17^, as shown in Fig. 1.

Microstrip array has a simple structure and easy fabrication proccess, which leads to compact and low-cost structures, but duo to its high losses in mmW frequency band the endfire antenna is recomanded to use^17,18^. Some types of passive power divider networks are Wilkinson, T-junction, and Resistive power divider. T-junction is lossless but it has two disadvanteges: un-matched at all ports and no isolation between output ports. The resistive type can be matched at all ports but it is lossy and doesn’t have isolation between output ports. But Wilkinson is lossless (if all ports are matched) and has good isolation.

**Figure 2:**
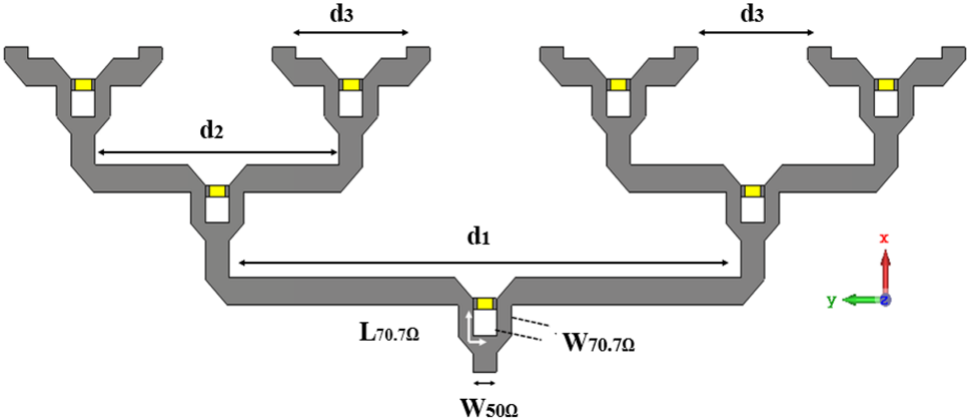
Eight way Wilkinson power divider.

**Figure 3:**
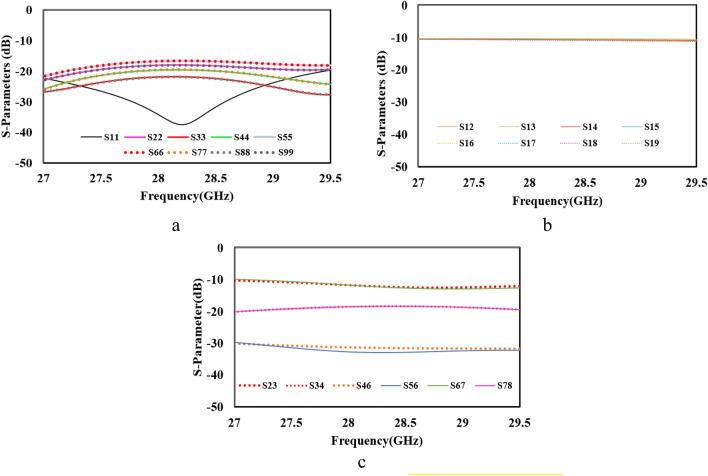
Simulation results of desinged eight-way WPD (**a**) reflection coeffcients, (**b**) insertion loss and (**c**) isolation.

In this paper Wilkinson Power Divider (WPD) has been adopted. To evaluate the WPD performance three parameters should be checked: reflection coeffcients, coupling and isolation between ports^18^. In two-way WPD, the isolation resistor is $2{Z}_{0}$ and the impedance of λ/4 is $\sqrt{2}{Z}_{0}$. For equal WPD (or 3 dB) the ${Z}_{0}=50\Omega$, the impedance of λ/4 is $\sqrt{2}{Z}_{0}=70.7\Omega$ and isolation resistor is ${2Z}_{0}=100\Omega$^18^. To design WPD at 28 GHz the TXline calculator is used. The substrate is Rogers RT/Duriod 5880 with 0.38 mm thickness, loss tangant of 0.0009 and relative permittivity of ${\varepsilon }_{r}=2.2$. The values for WPD are obtained as: W_50Ω_ = 1.18 mm, W_70.7Ω_ = 0.65 mm and L_70.7Ω_ = 1.97 mm (Fig. 2). The isolation resistor is *100Ω* (size is 1 × 0.5 mm^2^) from 0402 SMD family. For eight-way WPD, three stages of two ways WPD is needed. As shown in Fig. 2, d1 and d2 are approximately 4 times and 2 times longer than d_3_, respecivly. The distance between two output ports (d_3_) is about $\frac{\lambda }{2}$ to satisfy the array considerations. The performance of the eight-way designed WPD has been shown in Fig. 3. As it can be seen reflection coeffcient, isolation and insertion loss are in acceptable range and the observed deviation from the theoritical values are due to high frequency range of operation which leads to higher microstrip line loss (conductor, dielectric and radiation losses)^18–20^.

### Single elements

**Figure 4:**
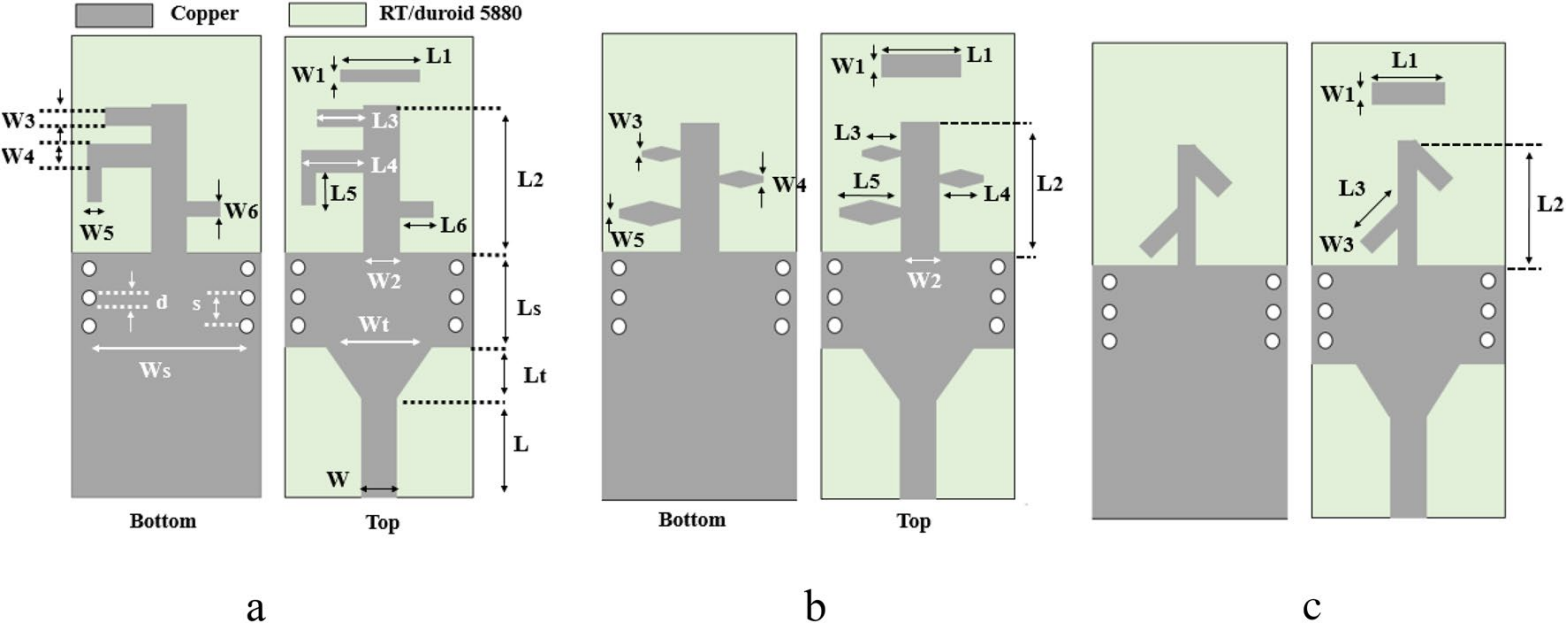
Structure of the proposed single element (**a**) antenna1, (**b**) antenna2 and (**c**) antenna3^21^.

The design procedure of three different single elements is completely described in^21^. Figure 4 shows the structures. The substrate is RT/duriod 5880 with 15mil thickness, ${\varepsilon }_{r}=2.2$ and ${\tan}\delta =0.0009$. The dimension of antenna1 according to Fig. 4a are *L*_*1*_ = 2.5 mm*, L*_*2*_ = 5 mm *L*_*3*_ = 1.615 mm*, L*_*4*_ = 2.275 mm*, L*_*5*_ = 1.25 mm*, L*_*6*_ = 1.125 mm*, W*_*1*_ = 0.4 mm*, W*_*2*_ = *1.2* mm*, W*_*3*_ = 0.5 mm*, W*_*4*_ = 0.75 mm*, W*_*5*_ = 0.5 mm*, W*_*6*_ = 0.5 mm. For antenna2 the dimensions in Fig. 4b are *L*_*1*_ = 2.8 mm*, L*_*2*_ = 4.22 mm *L*_*3*_ = 1.068 mm*, L*_*4*_ = 1.425 mm*, L*_*5*_ = 1.9 mm*, W*_*1*_ = 0.75 mm*, W*_*2*_ = 1.25 mm*, W*_*3*_ = 0.14 mm*, W*_*4*_ = 0.1875 mm*, W*_*5*_ = 0.25 mm. In addition, for the last one in Fig. 4c the dimensions are *L*_*1*_ = 2.5 mm*, L*_*2*_ = 3.9 mm *L*_*3*_ = 1.8 mm*, W*_*1*_ = 0.5 mm*, W*_*2*_ = 0.6 mm*, W*_*3*_ = 0.6 mm*.* For the feeding part (which is the same for all antenna) the calculated parameters are *Ls* = 3.5 mm*, wt* = 3.2 mm*, L* = 3.5 mm*, w*_*1*_ = 1.2 mm*, w* = 5.5 mm*, Lt* = 1.6 mm*, d* = 0.6 mm*, s* = 1.2 mm*.* The details of the design procedure for each of the single elements and the results (simulation and fabrication) of them are reported in^21^. All of these antennas have end-fire patterns and acceptable measurmant performance but are not applicable in 5G systems due to low gain values as the single element. Moreover, regarding the Ferris equation, the path loss become higher as the frequency increases. Accordingly, to overcome the path loss in 5G mobile communication system, minimmum value of 12 dB gain is required^22^. So, the antenna array configuration is proposed to achieve the required gain value.

### Linear array antennas

**Figure 5:**
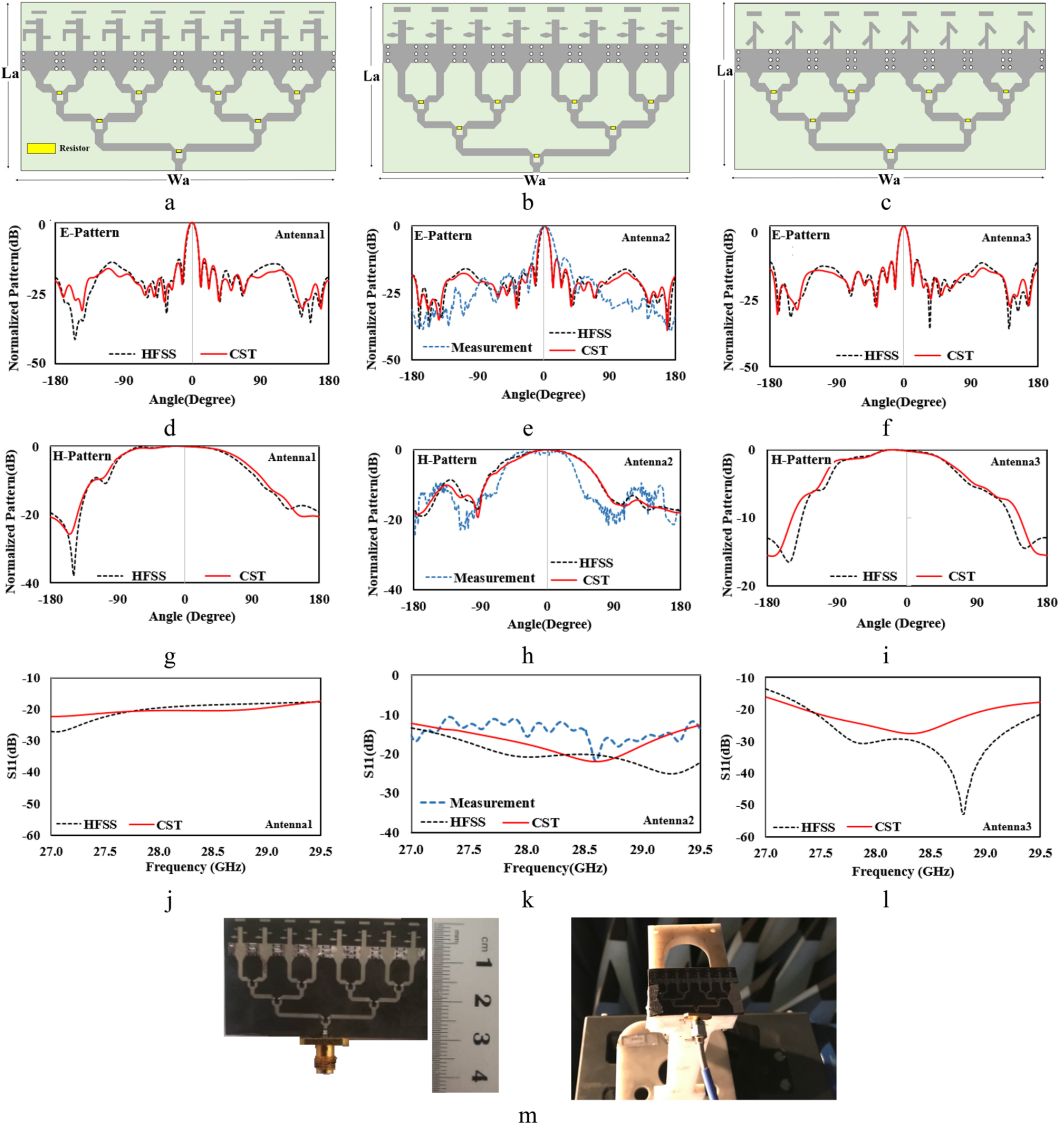
The array structure and simulation results of antenna1 (**a**,**d**,**g**,**j**), antenna2 with measurment results (**b**,**e**,**h**,**k**) and antenna3 (**c**,**f**,**i**,**l**) and (**m**) the prototype of proposed antenna2 with SMK connector.

Generally, the number of antenna array elements are 2^N^ owing to 2^N^-way is beneficial structure for designing a power divider with minimum losses. In addition, impedance matching can be accomplished easily^23^. The schematics of three different array antennas are shown in Fig. 5a–c. For better evaluation of array performance, two full-wave softwares (CST and HFSS) is used for simulation and the results of each array is shown in Fig. 5, respectively.

**Figure 6:**
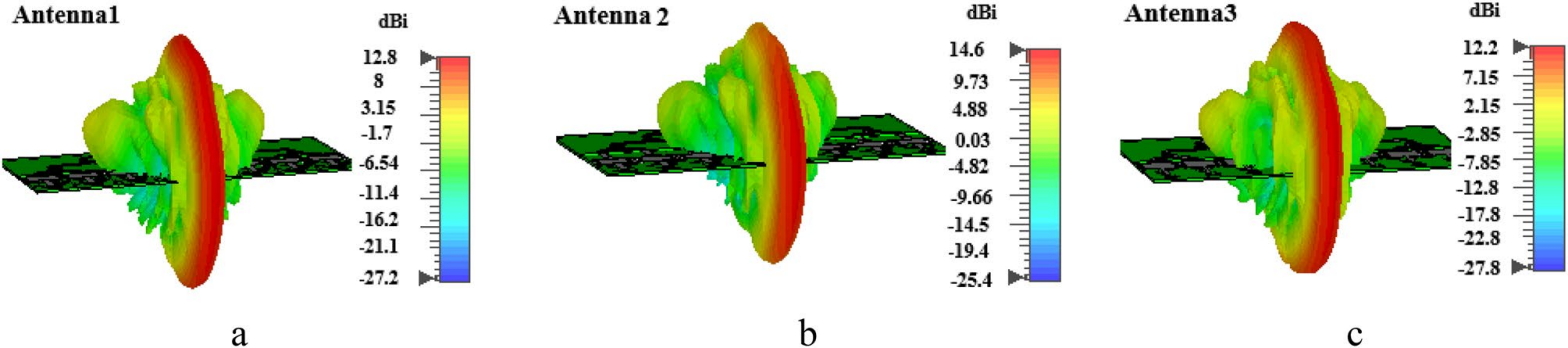
The 3D radiation pattern and calculated gain value of: (**a**) antenna1, (**b**) antenna2 and (**c**) antenna3.

As it can be observed, there is a good consistency between the simulation results in both softwares. Between these three proposed antennas, antenna2 is chosen to be fabricated and tested as shown in Fig. 5m. The connector that is used is SMK whith frequency range up to 40 GHz. So, in Fig. 5, the measurment results are shown for antenna2, too. As it can be seen, the measurment and simulation results for antenna2 are in good agreement, which suggests that the other two antennas are also applicable in this frequency band. The diffrerences between measurement and simulation results can be considered due to substrate and specially connector losses. It is obvious that performance in high frequency range duplicates the radiation and thermal loss effect of soldiering and SMD resistors. Moreover the loss and errors of fabrication and measurement devices can not be ommited. In Fig. 6, the simulated gain values are shown which are high enough for handheld 5G systems. Moreover, the endfire pattern of the proposed structures is suitable for 5G frequency bands because of its capabilitty (of array antenna) to consenpate the path loss. As will be discused in next section, directive antenna is a solution to minimize the SAR values in human tissue.

The size of three proposed structures are presented in Table 1 which shows acceptable reflection coeffcient and gain values while keeping the overall size as minimum as possible as a good candidates for handheld 5G systems The gain values are 12.85 dB, 14.6 dB, and 12.2 dB for antenna1, antenna2, and antenna3 respectively.

**Table 1:** The size of the proposed antennas.

Parameter	Size (mm^3^)
Antenna 1	26.42 × 53.6 × 0.38
Antenna 2	29.52 × 52 × 0.38
Antenna 3	26.42 × 52 × 0.38

## SAR assessments of proposed array antennas

The 5G systems have many interesting advantages, such as higher bandwidth and data rate; hence, they are growing surprisingly in the world. However, their probably adverse effects on human body tissues from such electromagnetic sources should appraise to ensure safety of human body. Some biological effects of electromagnetic fields such as cancer, blood brain barrier, the brain tumor, Cataract, skin disease, sleep disorder have been reported^24–27^. The other effects of mmW frequency are genotoxicity (DNA damage), cell proliferation, gene expression, cell signaling, electrical activity, and membrane effects have been briefed in^28^. Many references can be cited in which the advantages and disadvantages of SAR and PD parameters are discussed. Some of these references prefer SAR and the others PD. It seems that this issue is still an open subject, which needs to be investigate more carefully. Owing to the following reasons in this paper, the SAR is chose. According to FCC, the power density (PD) unit is used for the distances of 5 cm or more. Therefore, it only deals with far-field exposures and does not consider the near field exposures. On the other hand, some of the mmW devices such as handsets or tablets use almost near to the head, hand or in the pocket next to the human body (in a few millimeters distances i.e. near field region) and in these conditions, the PD is not a suitable unit to evaluate the human safety. In addition, estimations based on PD do not describe the absorbed power and distributed field, but only exhibit the travelling wave in human tissues. Hence, the SAR technique is used to study^13,29–32^.

There are some limitations to assess the SAR value in human body, because the adverse biological effects may occur, so the numerical simulations are to be used for SAR evaluation. SAR is a unit to determine the rate of how much energy from electromagnetic source is absorbed per mass unit by human tissues as show in Eq. (1).

1 $$SAR=\sigma \frac{{E}_{i}^{2}} {\rho } (W/Kg)$$

where $\sigma$ is the conductivity of tissue in unit (S/m), *E* is the electric field intensity in unit (V/m), $\rho$ is the mass density of tissue in unit (kg/m^3^). The SAR averages either over the whole body, or over a small sample volume (typically 1 g or 10 g of tissue). The unit of SAR is watt per kilogram^21^. SAR limits in International Commission on Non-Ionizing Radiation Protection (ICNIRP) and the IEEE C95.1–2019 standards is 2 W/kg over 10 g and according to FCC standard SAR limit for 1 g is 1.6 W/kg. These limits are for the frequencies up to 10 GHz and 6 GHz respectively. The SAR limits above these two frequencies for near field exposures at mmW have not been proposed yet which is due to near field exposure at mmW. However, it is an important topic to study.

### Head and handset model

**Figure 7:**
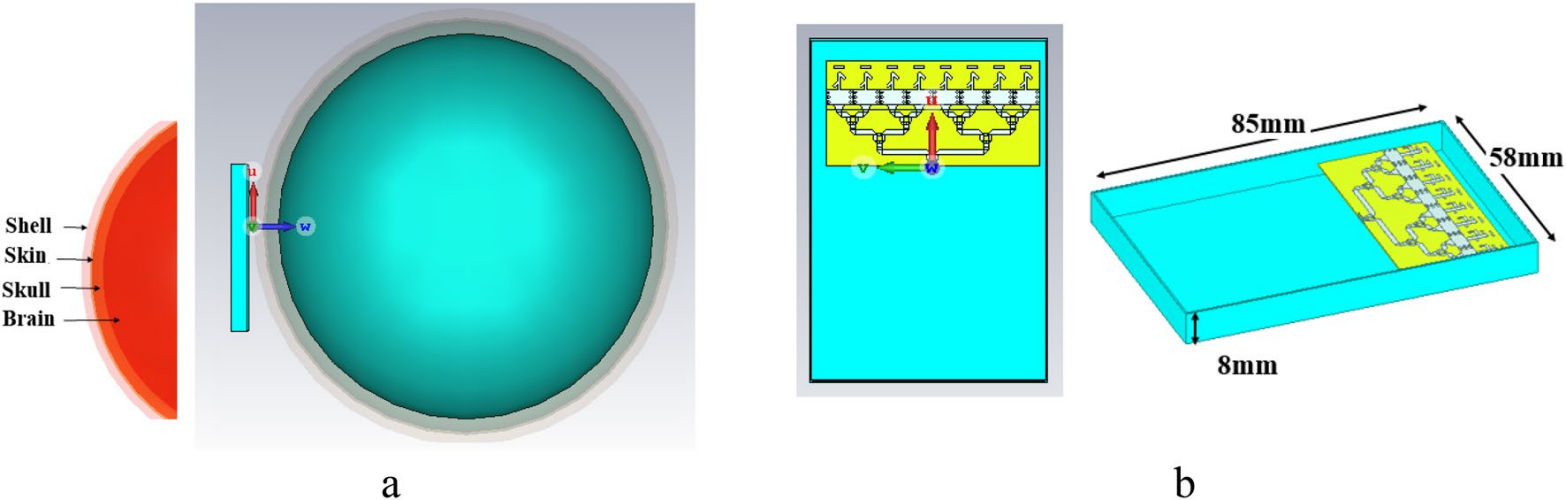
(**a**) Human head and handset model. (**b**) The handset dimension.

To simulate the SAR parameter, a three-layer spherical human head model including skin, skull, and the brain is situated near the antenna as an exposure source. All human tissues have different permittivity (${\varepsilon }_{r}$) and conductivity ($\sigma$), and their properties depend on many parameters such as frequency, age, etc. At 28 GHz, the properties and radius of three layers are listed in Table 2^3,21^. The covering shell is a low loss dielectric with relative permittivity of ${\upvarepsilon }_{r}=4.5$. The human head and handset model are shown in Fig. 7.

**Table 2:** Human head model properties at 28 GHz.

Tissues	${\varepsilon }_{r}$	$\sigma$ (S/m)	$\rho \; (\text{kg}/{\text{m}}^{3}$)	Radius (mm)
Skin	18.71	26.19	1100	106.5
Skull	7.51	8.88	1990	105.1
Brain	18.59	21.86	1041	98.3

**Figure 8:**
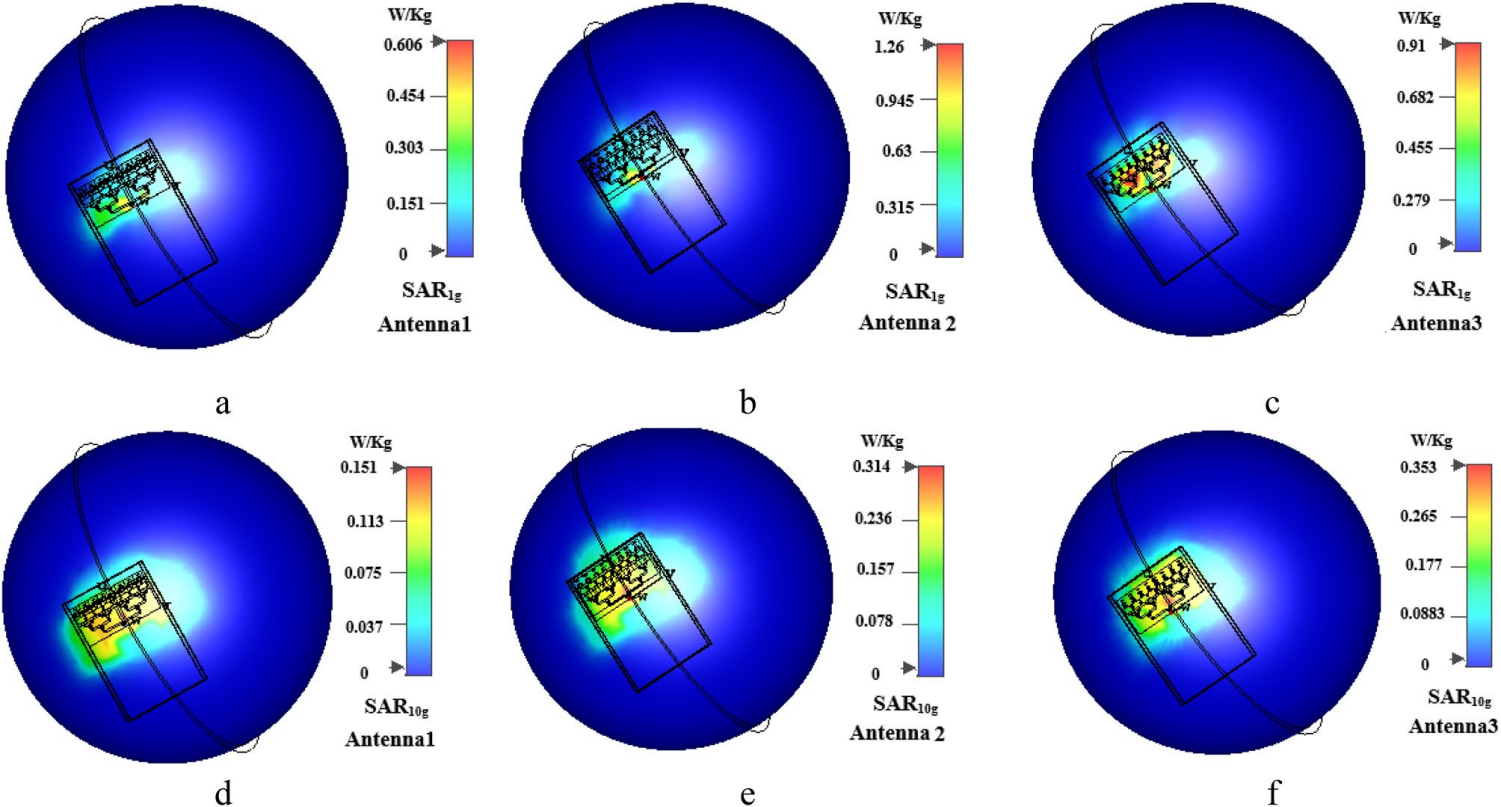
Simulated SAR parameter of antenna1 (SAR_1g_ (**a**), SAR_10g_ (**d**)), antenna2 (SAR_1g_ (**b**), SAR_10g_ (**e**)) and antenna3 (SAR_1g_ (**c**), SAR_10g_ (**f**)).

For the handset a plastic housing box with 58 × 85 × 8 mm^3^ dimension is used with ${\varepsilon }_{r}=3$ and $\sigma =0.02 \; \text{S}/\text{m}$ and 1 mm thickness in which the proposed antenna array is placed on top^33^. A glass with ${\varepsilon }_{r}=5.5$ used too as a screen of the handset^8^. The input power for the antennas in 5G systems can be set to 15 dBm, 18 dBm and 20 dBm according to FCC^34^ and the distance between head and antenna are 5 mm^3,13,34^. Figure 8. shows the results of SAR_1g_ and SAR_10g_ for 15 dBm. From this figure, it can be observed that: (1) The SAR at the nearest distance from antennas are more than others are. (2) The SAR_1g_ is higher than SAR_10g_. (3) By increasing the distance between antenna and human head model the SAR is decreased.

Table 3 shows the simulation results of SAR_1g_ and SAR_10g_ of three proposed array and single antennas with 15 dBm power. The single element SAR values in our published paper^21^ are used in Table 3. As can be observed, all of the simulated values are under the FCC and ICNIRP standard limits for array antennas too. Considering the same feeding part for three proposed structures and different radiating element for each, the antennas have different electric field strength (E) which leads to different SAR values according to Eq. (1).

**Table 3:** The averaged SAR_1g_ and SAR_10g_ for 15 dBm input power.

Antenna array	SAR (W/kg)	Dipole antenna	SAR (W/kg)	Single element	SAR (W/kg)	Dipole antenna	SAR (W/kg)
Ant.1(SAR1g)	0.606	SAR_1g_	1.81	Ant.1(SAR1g)	0.236	SAR_1g_	1.81
Ant.2(SAR1g)	1.26	–	–	Ant.2 (SAR1g)	0.284	–	–
Ant.3(SAR1g)	0.91	–	–	Ant.3 (SAR1g)	0.416	–	–
Ant.1(SAR10g)	0.151	SAR_10g_	0.51	Ant.1 (SAR10g)	0.081	SAR_10g_	0.51
Ant.2(SAR10g)	0.31	–	–	Ant.2 (SAR10g)	0.071	–	–
Ant.3(SAR10g)	0. 353	–	–	Ant.3 (SAR10g)	0.169	–	–

Although all results for antenna array are larger than single elements. It can be cause by more radiation elements in array type. In the commercial SAR measurement system, a diploe antenna is used to measure the SAR parameter. For better comparison, the SAR values of these antennas i.e. proposed antennas, and dipole antenna (from^21^) at 28 GHz are shown in Table 3. The results of both array and single element SAR values are lower than dipole antenna, which shows that proposed end-fire (directive) antennas have lower SAR rather than common dipole antenna. In Table 4 the simulation results with 20 dBm input power are also presented. It can be seen that the values are lower than standard limits and dipole antenna too.

**Table 4:** The averaged SAR_10g_ for 20 dBm input power.

Array antenna	SAR (W/kg)	Dipole antenna	SAR (W/kg)
Ant.1 (SAR10g)	0.478	SAR10g	1.62
Ant.2 (SAR10g)	0.998		–
Ant.3 (SAR10g)	0.92		–

It is well known that electromagnetic fields can damage human tissues, thus designing the low SAR antenna is desirable for mobile devices such as handsets, which use in human body vicinity to reduce probable adverse health effects. In fact, by decreasing the SAR, the field penetration in the human tissues will decrease.

To compare the results of the SAR values and performance of three array antennas Tables 5 and 6 are provided. From the Table 5 the SAR values for proposed antennas are almost lower than the other works at 28 GHz and all of them are low SAR. From the Table 6 the proposed antennas are smaller than other references and all of them have enough gain for 5G systems.

**Table 5:** Comparison the SAR of three antenna performances with other 5G antenna references at 28 GHz.

Ref	d (mm)	Power (dBm)	Array	Gain (dB)	SAR (1 g)	SAR (10 g)
^13^	5 mm	24	2 × 2	11.23	1.35	–
^35^	N.A	N.A.	–	9.485	1.42	0.3
^22^	N.A	N.A.	2 × 2	12.3	–	0.37
^36^	N.A	24	MIMO	$\approx$ 10	–	Max:1.2
			8 × 8		–	Min:0.8
Ant. 1	5 mm	15	1 × 8	12.85	0.56	0.15
Ant. 2	5 mm	15	1 × 8	14.6	1.25	0.31
Ant.3	5 mm	15	1 × 8	12.2	0.91	0. 35

d: Distance between antenna and human head model.

**Table 6:** Comparison of three antenna results with other references of 5G.

`Ref	Antenna type	Substrate	BW (dB)	Relative size (${\lambda }_{0}^{3})$	Array	Gain (dB)	Size reduction
^2^	Phase array	Rogers 5880	28	N.A	1 × 8	11	–
^7^	Quasi Yagi	N.A	31–34	3.97 × 0.51 × 0.06	1 × 8	$\approx$ 15.5	35.7%
^9^	Vivaldi	Rogers 5880	24.5–28.5	5.61 × 2.8 × 0.047	1 × 8	11.2	56.57%
^37^	Vivaldi	Rogers RO4003	25–40	12.61 × 9.1 × 0.02	1 × 4	16	78.97%
^38^	Quasi-Yagi	Arlon Ad 350	25–27	8.41 × 3.73 × 0.35	1 × 8	10.5–12	95.6%
^23^	Vivaldi	ISOLA IS300MD	27.5–28.5	5.6 × 12.6 × 0.074	1 × 4	8.01	90%
^39^	5G	4 substrate	28	7.55 × 7.55 × 0.22	4 subarray	11.62	96.21%
*	Antenna 1	RT/Duroid 5880	27–29.5	2.46 × 5 × 0.036	1 × 8	12.85	–
*	Antenna 2	RT/Duroid 5880	27–29.5	2.8 × 4.86 × 0.036	1 × 8	14.6	–
*	Antenna 3	RT/Duroid 5880	27–29.5	2.5 × 4.86 × 0.036	1 × 8	12.14	–

## Conclusion

In this paper three compact, small size, low weight, and low SAR array antennas are designed. The feeding part of them is WPD. Owing to their good patterns and reflection coefficient at 27–29.5 GHz from simulation in CST and HFSS and the measurement data, they are suitable for applying in 5G systems. Since the human health effects from electromagnetic fields is very important subject and the user are worry about it, the SAR_1g_ and SAR_10g_ of the antennas at 15 dBm (and SAR_10g_ at 20 dBm) have been simulated in human head model. All the results are lower the standard limits.

The distance between antenna and human head model is 5 mm. Although using hands-free increase the distance and can reduce the SAR. To more examination, results of SAR are compared with dipole antenna that use in commercial SAR measurement system. One of the methods to reduce the SAR is using of directive antenna^40^. Since our proposed antennas are end fire (directive antennas), thus, the results of SAR are suitable. It is noted that in real SAR measurement systems it is impossible to model the human head model in the layers, because the human tissue equivalent material are in jell or liquid form. Therefore, it may be said that the commercial results are not accurate and more investigation for better tissue model is necessary.
